# Spectroelectrochemistry of Electroactive Polymer Composite Materials

**DOI:** 10.3390/polym14153201

**Published:** 2022-08-05

**Authors:** Oxana L. Gribkova, Alexander A. Nekrasov

**Affiliations:** A.N. Frumkin Institute of Physical Chemistry and Electrochemistry RAS, Leninskii Prospect 31, 119071 Moscow, Russia

**Keywords:** conducting polymers, electroactive polymers, polymer composites, spectroelectrochemistry, UV–Vis–NIR spectroscopy, Raman spectroscopy, infrared spectroscopy, electron spin resonance spectroscopy

## Abstract

In this review, we have summarized the main advantages of the method of spectroelectrochemistry as applied to recent studies on electrosynthesis and redox processes of electroactive polymer composite materials, which have found wide application in designing organic optoelectronic devices, batteries and sensors. These polymer composites include electroactive polymer complexes with large unmovable dopant anions such as polymer electrolytes, organic dyes, cyclodextrins, poly(β-hydroxyethers), as well as polymer-inorganic nanocomposites. The spectroelectrochemical methods reviewed include in situ electron absorption, Raman, infrared and electron spin resonance spectroscopies.

## 1. Introduction

Electroactive (EA) polymer functional materials have recently found wide application in designing organic and hybrid photovoltaic solar cells [1,2,3], organic light emitting diodes [4,5,6], electrochromic “smart windows” and displays [7,8,9], chemical [10] and biological [11] sensors. The main building blocks of numerous materials of this kind are aniline, thiophene, pyrrole and their derivatives. Accordingly, the most well-studied, but still popular among researchers, electroactive polymers are polyaniline (PANI), polythiophene (PTh, for example poly(3,4-ethylenedioxythiophene) (PEDOT)) and polypyrrole (PPy). They possess relatively low oxidation potentials, a high electrical conductivity in the doped state, transparency in the visible region, thermal and environmental stability in air and aqueous media, biocompatibility, and ease of synthesis.

In this regard, fundamental studies of synthesis processes, the study of spectroelectrochemical properties, and the mechanisms for the redox and acid doping of EA polymers are important trends in the world of science. Spectroelectrochemistry is a powerful tool to investigate the mechanisms of various structural transformations which occur during synthesis, redox doping/dedoping, sensing response and operation life degradation in EA polymers and their composites. 

Large dopant anions such as polymer electrolytes, phthalocyanines, organic dyes, cyclodextrins, poly(β-hydroxyethers), etc. have been successfully used in the electrochemical synthesis of EA polymers for more than 35 years. They play the role of a doping anion, electrolyte, and also serve as active components that affect the kinetics of electrosynthesis, as well as the structure and properties of the resulting polymer composites. Electrosynthesis of EA polymers in the presence of large dopant anions improved the surface uniformity, mechanical properties and thermal stability of the films. The large anion is included in the composition of the film and is not removed from it during dedoping. As a result, the mechanism of charge compensation during redox transformations in EA polymers changes. Replacing inorganic small molecules with large dopant anions enhanced the ambient stability of EA polymers due to their nonvolatile nature. 

The large conjugated system of dyes can exert electronic interaction with the π-system of the EA polymers, altering their electronic properties. The incorporation of organic dyes into the EA polymers enhanced the electrochromic properties such as optical contrast and color modulation [12,13,14]. Sulfonated dye-doped modified PPy film demonstrated a high sensitivity and good selectivity in chemiresistor gas sensor [15].

Cyclodextrins can bond to the electrode and acted as molecular templates to restrict the growth sites of EA polymers within the cyclodextrins cavities [16]. PPy film doped with Heparin polyanion enhanced electrochromic contrast and redox stability [17]. The sulfonated redox-active polycatechol, immobilized in PEDOT film, enhanced its energy storage capacity [18].

The use of sulfonated polyelectrolytes (PE) during electrosynthesis of EA polymers produces a templating effect leading to changes in their structure and properties (optical, electrochemical, spectroelectrochemical, morphology). Thin layers of PEDOT composites with PE of various structures and forms (acid or base) were used as transparent hole-transport layers in perovskite solar cells based on a CH_3_NH_3_PbI_3_ photoactive layer [19]. EA polymer (PEDOT, PPy, PANI) composites with polysulfonic acids of different structure and chain flexibility demonstrated perovskite optical sensing properties to ammonia gas [10].

Another important direction in the modification of EA polymers is the preparation of their composites with carbon materials and metal oxides. Incorporation of nanostructured inorganic compounds into the EA polymers combines valuable features of the organic and inorganic components in a single material and modifies the properties of the EA polymers, leading to the development of multifunctional devices [20]. The synthesis of nanostructured metal oxide–EA polymer composites lead to synergistic enhancement of their electrochromic and pseudo-capacitive properties [21]. Novel nanostructured carbon materials (nanotubes, graphene) can significantly improve the electrical conductivity and electrochemical performance of PEDOT in areas such as the stability of electrochromic characteristics, coloration efficiency, and switching time due to an important role of the electron transfer in the bulk of PEDOT films [22,23]. 

This review considers the general regularities of the spectroelectrochemistry of such typical representatives of the EA polymers as PANI, PPy, PEDOT, their derivatives and composites with inorganic materials, large organic anions, etc. The characteristic features of the spectroelectrochemical behavior of EA polymers are discussed in the example of composites of EA polymers with PE. Understanding the role of the large organic anions and inorganic materials, and its effect on the structure and properties of the obtained EA polymers composites, can help in the development of polymer layers with predetermined functionality and improved resource characteristics for various applications, such as electrochromic, electroluminescent, and photovoltaic devices, power sources, capacitors, chemical and biological sensors, etc.

## 2. Spectroelectrochemical Methods

The main advantage of using electrochemical control of the above-mentioned processes is the possibility to produce moderate stepwise influence on the system, which allows one to study in detail spectral characteristics, along with the electronic and chemical structure of all intermediate products. On the other hand, there are also high-speed spectroelectrochemical methods that make it possible to identify limiting stages and to determine the maximum possible operation speed for the functional material to optimize the operation parameters and prevent degradation due to overoxidation/overreduction.

Spectroelectrochemical methods are subdivided into the following cases depending on the physical method used to register changes in the state of system upon electrochemical influence: (1) spectroscopy in the ultraviolet, visible and near-infrared ranges (UV–Vis–NIR); (2) Raman spectroscopy; (3) infrared (IR) spectroscopy, Fourier transform infrared (FTIR) spectroscopy and FTIR in attenuated total reflection (ATR) mode; (4) electron spin resonance spectroscopy (ESR). In this review we will not consider electrochemical impedance spectroscopy since this is a purely electrochemical technique and there are numerous review publications in this field. 

### 2.1. In Situ UV–Vis–NIR Spectroscopy

UV–Vis–NIR spectroscopy allows one to trace changes in the electronic structure upon electrochemical influence during electrosynthesis and redox doping/dedoping. Concerning electroactive polymer materials, the UV–Vis–NIR spectra measured in situ during electrochemical oxidation/reduction give information about doping/dedoping processes which are accompanied by the appearance of new electron energy levels within the bandgap of a polymer. It is important to emphasize that charged (electron-deficient or electron-excessive) fragments of the polymer chain serve as the dopant in this case. To maintain electroneutrality, the arising charged fragments should be compensated by the insertion/expulsion of ions of the opposite sign (often called counterions) into/from the polymer film. Nevertheless, still there are numerous publications [24,25,26,27] which argue that electroactive polymers are doped by the counterions (called doping ions), which is obviously not the case. This statement is supported by the results of the group of MacDiarmid [28,29], who observed an appearance of the short-lived spectrum of PANI radical cations upon photoexcitation of the base form of emeraldine in dry films. Considering counterions, their diffusion/migration rate inside the polymer film obviously influences the rate of oxidation/reduction processes, but the limiting factors in this case are propagation of charged species along the polymer chains and conformational changes in the polymer chain due to this charging.

One can only speak about some kind of doping anions if these are huge, unmovable (some of them of PE nature [30,31]) anions trapped inside the electroactive polymer film during the synthesis. Purposeful choice of such PE dopants will help one to modify physico-chemical properties and morphology of the film and influence transport of movable charge-compensating inorganic counterions in the bulk of the film. 

The method of UV–Vis–NIR spectroelectrochemistry is based on the combination of the Faraday’s and Beer’s laws:

*m* = *QM*/*nF *and* A *= *εcl* (1)

where *m*—mass of electrogenerated substance; *M*—molecular mass; *Q*—charge consumed in the electrochemical reaction; *n*—number of electrons participating in the electrochemical reaction; *F*—Faraday’s constant (96500 C mol^−1^); *A*—optical absorbance of electrogenerated substance at the maximum of its absorption band; *ε*—molar extinction coefficient of electrogenerated substance; *c—*molar concentration; *l*—thickness of light-absorbing layer.

By combining these two equations one can obtain:

*A* = *Qεl*/*nFV* or *A* = *Qε*/*nFS* (2)

where *V*—the volume of light-absorbing layer and *S*—its surface area (usually electrode surface area). So, assuming the validity of Faraday’s and Beer’s laws, the absorbance is directly proportional to electrochemical charge.

There is one more valuable modification of the Equation (2):

*dA* = *dQε*/*nFS* or *dA* = *iε*/*nFS* (3)

where *i*—the current flowing during electrochemical process. So, the instant change in the absorbance is directly proportional to the electrochemical current. Accordingly, one can get additional valuable information by comparing the cyclic voltammetry (CV) curves (current vs. potential, *i = f*(*E*)) and the dependences *dA = f*(*E*). The latter method is usually called spectrovoltammetry [32] or derivative cyclic voltabsorptometry (DCVA) [33] and its application to study electroactive polymers will be discussed below in more details.

#### 2.1.1. Spectra at Fixed Potentials

Registration of UV–Vis–NIR spectra at fixed potentials allows one to study long-lived intermediate products of the oxidation/reduction process. These experiments may be conducted both in the transmission (absorbance) and reflection modes. In the transmission (absorbance) mode one has to use so-called optically transparent electrodes (OTE). Usually, three types of OTE are used: (1) glass or polymer film substrates covered by conducting oxide films such as fluorine doped tin oxide (FTO) [34] or mixed indium-tin oxide (ITO) [35]; (2) free-standing lithographic-galvanic (LIGA) fabricated metal microgrids (LIGA-electrodes [36]); (3) metal microgrids (micromesh) formed on the surface of polymer (mainly polyethylene terephthalate) substrate [37]. In the latter two cases it is important to ensure that the gap in microgrid cells is completely closed by either electrodeposited or solution-cast film of EA polymer. In spite of the latter complication, the spectroelectrochemical results obtained on microgrid OTEs are far more informative, particularly in the NIR range, than conducting oxides, which have rather high absorbance and allow one to combine spectroelectrochemical and in-situ electron spin resonance studies [38]. 

The following examples demonstrate the advantages of using UV–Vis-NIR spectra registered at fixed potentials for the interpretation of the electronic structure of composites of PANI with polymeric sulfonic acids of different chemical structures.

**Figure 1:**
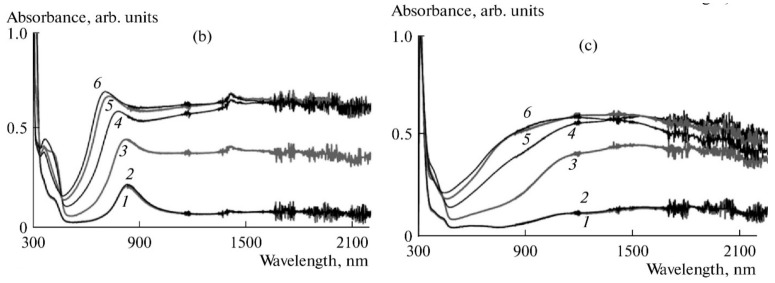
Evolution of in situ UV–Vis–NIR spectra of PANI films prepared by electrochemical polymerization in the presence of flexible-chain PAMPSA (**b**) and rigid-chain t-PASA (**c**) polyacids with potential, V: 0.0(1), 0.2(2), 0.4(3), 0.6(4), 0.8(5), and 0.9(6). Electrolyte: 0.1 M HClO_4_ and 0.9 M NaClO_4_ in acetonitrile. Reprinted by permission of Springer Nature Customer Service Centre GmbH: Springer Nature, Russian Journal of Electrochemistry, “The spectroelectrochemical behavior of films of polyaniline interpolymer complexes in the near infrared spectral region”, A. A. Nekrasov et al., Copyright 2012 [39].

Figure 1 shows the evolution of in situ UV–Vis–NIR spectra with potential for PANI films prepared by electrochemical polymerization in the presence of flexible-chain (poly-2-acrylamido-2-methyl-1-propanesulfonic acid (PAMPSA)) and rigid-chain (poly-4,4′-(2,2′-disulfonic acid)-diphenyleneterephthalamide (t-PASA)) polyacids [39].

It is clearly seen that the composite film of PANI with the rigid-chain polyacid absorbs preferentially in the NIR range, while the spectra of the complex with PAMPSA look very much like the spectra of conventional PANI. The most possible reason is that the flexible-chain PAMPSA is able to adopt its conformation easily for more efficient compensation of charged species in PANI chains during the electropolymerization, resulting in a less distorted intermolecular structure of the film. As a result, one can observe the whole set of transitions between PANI redox forms: leucoemeraldine–radical cations–localized polarons–pernigraniline. On the contrary, the electrosynthesis in the presence of rigid-chain polyacid leads to the formation of distorted structure, in which the rigid polyacid molecules cannot fully compensate all charged species in PANI macromolecule, resulting in the retarded formation of quinoid structures (pernigraniline). At the same time, the film of the PANI-t-PASA composite exhibits more efficient electrochromic modulation of the NIR range, which may be very useful in some applications. 

**Figure 2:**
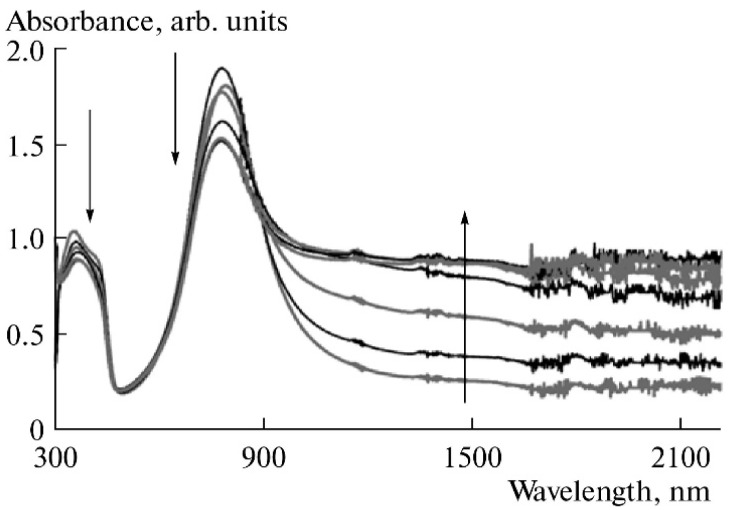
Evolution of the spectrum (at 0.6 V) of the film of the chemically synthesized PANI–PAMPSA interpolymer complex during CV cycling in the range of potentials of the first redox stage in an acetonitrile solution of 0.1 M HClO_4_ and 0.9 M NaClO_4_. The number of cycles increases in the direction of the arrows: 0, 3, 6, 9, 12, 15, 18. Reprinted by permission from Springer Nature Customer Service Centre GmbH: Springer Nature, Russian Journal of Electrochemistry, “The spectroelectrochemical behavior of films of polyaniline interpolymer complexes in the near infrared spectral region”, A. A. Nekrasov et al., Copyright 2012 [39].

Figure 2 shows the evolution of in situ UV–Vis–NIR spectra of the PANI-PAMPSA composite film, which was prepared by casting an aqueous solution of the polymer composite obtained by chemical oxidative polymerization, in the course of continuous potential cycling within the range of potential of the first oxidation/reduction stage of PANI [39].

If one compares the initial spectrum (0 cycles) in the Figure 2 with the corresponding spectrum in the Figure 1b (spectrum 4) it is easy to see that the electrodeposited film exhibits far more intensive absorption in the NIR range. This is due to the fact that the electrodeposited film is growing from the electrode surface maintaining electric contact between the PANI chains. As a result, the degree of charge carriers’ delocalization in this case is higher than in the case of solution-cast film, in which PANI chains are partly isolated from each other by non-conductive polyacid macromolecules. As the number of CV cycles increases, one can observe a gradual increase in the NIR absorption and a corresponding decrease in the absorption of localized polarons near 800 nm, which is accompanied by the growth of CV redox charge. This may be explained by intensive conformational changes in PANI macromolecules during redox transformations (bent-chain leucoemeraldine–flat-chain emeraldine) resulted in the establishing of a better contact between isolated PANI chains.

Another valuable spectroelectrochemical technique is the registration of difference spectra at fixed potentials [40]. In this case one takes some of the system’s states (for example, the reduced state) for a background, measures the spectrum of this state and stores it in the spectrometer memory. Then one measures all other spectra at fixed potentials against this background. The resulting spectra contain both positive and negative amplitudes corresponding to the species consumed or generated, respectively, during the oxidation. This allowed the authors to identify clearly individual absorption components of the spectrum of two thiophene derivatives used in organic field-effect transistors.

#### 2.1.2. Deconvolution of UV–Vis–NIR Spectra

As can be seen from the above-presented figures, the spectra of PANI (and most of conducting electroactive polymers) demonstrate no well-resolved absorption bands, this fact being one of the consequences of arising chain conjugation and delocalization of the charge carriers. This is due to the existence of a wide range of electronic states, whose shares in the resulting spectrum are changing during redox transformations. There are several methods for deconvolution (peak fitting) of complex electronic absorption spectra, including use of Gaussian and/or Lorentzian approximations of the shapes of individual absorption bands [41] and Matrix Rank Analysis [42]. However, the number and shapes of the approximated individual absorption bands used for this deconvolution are usually chosen in a voluntary way. Fortunately, there is a simple and intuitive Aletsev-Fok method free from these drawbacks, which we have first applied for the deconvolution of PANI spectra measured at fixed potentials [43]. To apply this method, one need to have a set of the absorption spectra that differ by contributions of individual absorption bands; these bands must not overlap completely (when one band screens another, narrower band). The number of such spectra must be equal to or greater than that of predicted spectrum components (the more the better).

**Figure 3:**
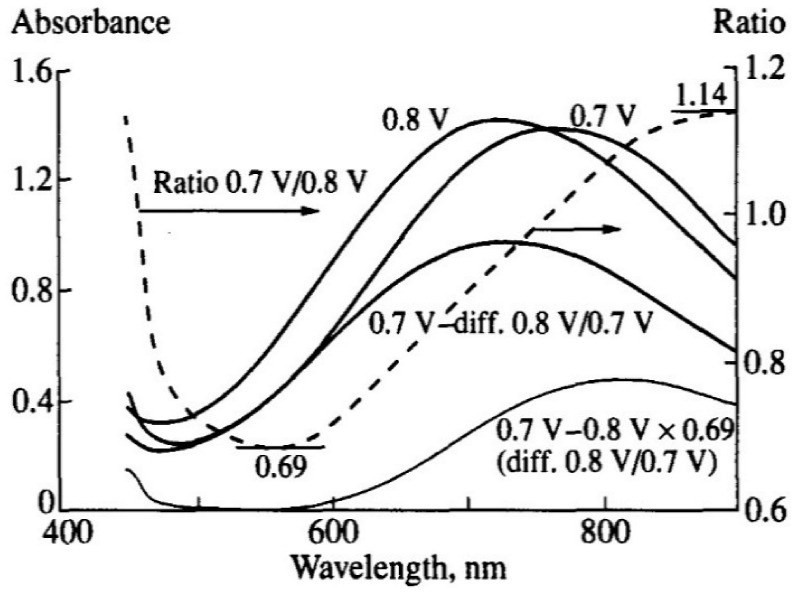
Demonstration of the principle of the Alentsev-Fok method for deconvolution of electron absorption spectra of PANI recorded in 1M aqueous HCl at 0.7 and 0.8V. Reprinted by permission from Springer Nature Customer Service Centre GmbH: Springer Nature, Russian Journal of Electrochemistry, “Isolation of Individual Components in the Electronic Absorption Spectra of Polyaniline from the Spectroelectrochemical Data”, A. A. Nekrasov et al., Copyright 2000 [43].

Figure 3 [43] explains the main principle of Aletsev–Fok method: if, in a certain spectral range, two spectra contain the different contributions of an individual absorption band, and this band does not overlap with other absorption bands, the ratio of absorbances of these two spectra is constant in this spectral range. 

As seen in Figure 3, the ratio of the two spectra (dashed line) has two plateaus: near 550–580 nm and near 890–900 nm. For example, the ratio value for the plateau near 550–580 nm is 0.69. In the next step one should multiply each value of the absorbance in the spectrum recorded at 0.8 V by 0.69 and subtract the result from each point of the spectrum recorded at 0.7 V. As a result, we obtain a zero absorbance in the spectral range where the corresponding individual component of the spectra absorbs.

**Figure 4:**
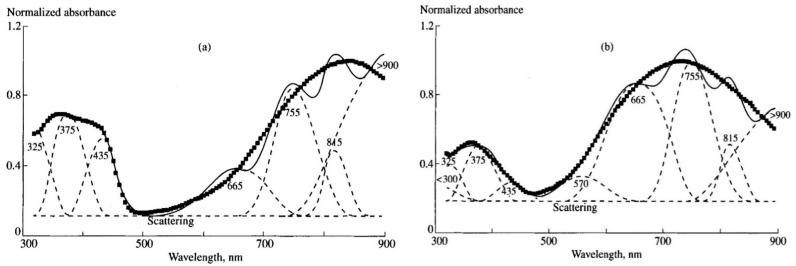
The results of deconvolution of PANI absorption spectra recorded in 1M aqueous HCl at 0.4 (**a**) and 0.8 (**b**) V. Reprinted by permission from Springer Nature Customer Service Centre GmbH: Springer Nature, Russian Journal of Electrochemistry, “Isolation of Individual Components in the Electronic Absorption Spectra of Polyaniline from the Spectroelectrochemical Data”, A. A. Nekrasov et al., Copyright 2000 [43].

By applying this procedure to the spectra measured at different potentials, as well as to the difference spectra derived from the deconvolution procedure, one can gradually decrease the number of absorption bands in the spectra till the moment when there is only one left. This will be truly individual band. Figure 4 shows the results of deconvolution of PANI absorption spectra recorded at 0.4 and 0.8 V.

Deviations between the experimental and fitted spectra in the long wavelength range are due to insufficient accuracy of the calculations of the asymptotic edges of the individual bands. To overcome this drawback, one can approximate the resulting individual absorption bands by a linear combination of Gaussian and Lorentzian functions, which will increase accuracy at the edges. As distinct from simple Gaussian/Lorentzian deconvolution of the full spectrum, the approximation the individual absorption band gives real information about its shape.

Based on the literature data and our later results [44] (which will be discussed below) the deconvoluted absorption bands were attributed to different electron transitions in PANI (nm): <300 — π-π* transitions in benzenoid or quinoid rings for leucoemeraldine or pernigraniline forms of PANI, respectively; 325 — π-π* transitions in benzenoid rings for emeraldine form of PANI; 375—not assigned; 435—radical cations; 570—exciton absorption in quinoid rings; 665—dimers of radical cations; 755—localized polarons; 815—not assigned; >900—absorption of free charge carriers.

#### 2.1.3. Derivative Cyclic Voltabsorptometry (DCVA)

As was mentioned above (Equation (3)) the first derivative of absorbance changes on time is some analog of current (one may call it “optical current”) flowing through the spectroelectrochemical cell. Therefore, the dependence of *dA* on time or potential may be compared with the corresponding dependency of the current in potentiostatic or potentiodynamic processes, correspondingly, giving valuable information about the capacitive components of the current and interconversion of different structural forms of the electroactive polymer. DCVA method was first applied to study PANI [45] and poly(3-methylthiophene) [46] and was mentioned in the latter paper as “spectrovoltammetry”. However, the term DCVA was earlier proposed in [47]. 

The problem arises: how to choose wavelengths at which one need to monitor absorbance changes? The visible maxima in the spectra sometimes are composed of two or three individual absorption bands. To solve this problem, we first have performed DCVA experiments [44] at the wavelengths corresponding to individual absorption bands separated from the spectra of PANI using Alentsev-Fok method. 

**Figure 5:**
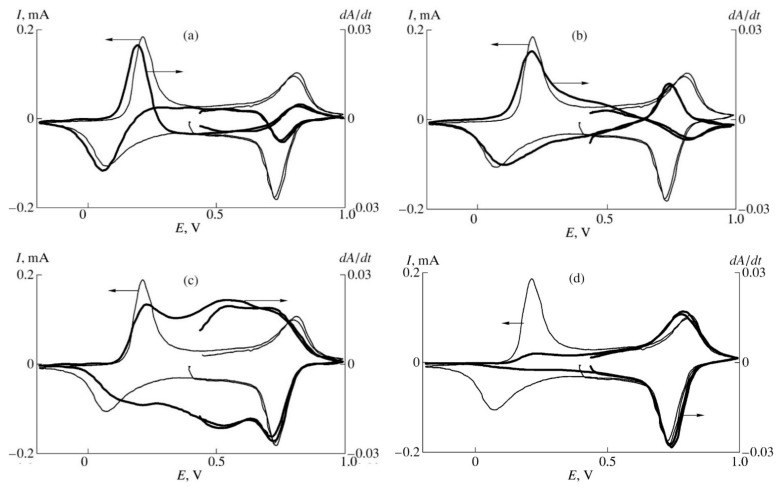
DCVA (thick lines) and CVA (thin lines) curves for PANI films measured in HClO_4_ at (**a**) 435, (**b**) 755, (**c**) 665, and (**d**) 570 nm. Potential sweep rate is 10 mV/s. Reprinted by permission from Springer Nature Customer Service Centre GmbH: Springer Nature, Russian Journal of Electrochemistry, “On the Role Played by Dimers of Radical Cations in the Process of Electrochemical Oxidation–Reduction of Polyaniline: The Data that Were Obtained Using the Method of Cyclic Voltabsorptometry in the Presence of Counteranions of a Diverse Nature”, A. A. Nekrasov et al., Copyright 2004 [44].

Figure 5 shows the DCVA results for PANI film measured in 1M aqueous HClO_4_ at different wavelength, corresponding to: 435 nm—radical cations; 570 nm—quinoid fragments; 665 nm—dimmers of radical cations; 755 nm—localized polarons.

As one can see from the figures above, the DCVA peak of radical cations in the first oxidation stage precedes in the potential scale the corresponding peak of current being actually situated at the front of the peak of current. Importantly, the same situation was observed by Genies and Lapkowski [48] for the peak of spin concentration during in situ ESR studies. The existence of second positive a DCVA peak of radical cations in the range of potentials of second oxidation stage of PANI is also supported by the ESR results in [48]. This correspondence between DCVA and ESR results was achieved exclusively thanks to the use of the wavelength 435 nm determined from Alentsev-Fok deconvolution, because similar studies using visually determined absorption peak position for radical cations showed complete matching of the DCVA and current peaks position [45].

The DCVA peaks for localized polarons and quinoid structures correspond to the first and second current peaks. Surprising but quite reasonable results were obtained in the spectral range near 665 nm: the maximum of DCVA signal is situated in the intermediate range of potentials between the first and the second oxidation stages of PANI. It is important that this signal cannot be obtained by linear combination of the signals measured at 755 and 570 nm. Therefore, this result confirms the supposition that the spectral range near 665 nm corresponds to dimerization of the radical cations by a chemical process, consuming no current.

#### 2.1.4. Color Impedance Spectroscopy

There is one more dynamic spectroscopic technique, which allows one to exclude the influence of capacitive components of electrochemical current on the interpretation of results. It is called color impedance and was first proposed in Fujishima laboratory [49]. It is based on the registration of frequency-dependent absorbance changes at small oscillations of potential. Further processing of the data is handled in the same manner as the electrochemical impedance data. In this method the proper choice of spectral range, in which one should register absorbance changes, is also a matter of particular significance. Later [50,51] color impedance was coupled with electrochemical impedance and electrochemical quartz crystal microbalance to study electroactive polymers.

### 2.2. In Situ Raman Spectroscopy

The next popular spectroelectrochemical technique, which allows one to trace structural transformations during electrosynthesis and redox doping/dedoping of EA polymers, is Raman spectroscopy. These experiments are usually performed in situ on metallic (Au [52], Pt [53]) or glassy carbon [54] electrodes. At the same time, one should take into account that during the Raman experiment on reflective electrodes, the incident laser beam passes several interfaces, where absorption, refraction and reflection, as well as interference of the incident and reflected beams, may occur. These interfaces are: (1) air/glass (the wall of the spectroelectrochemical cell); (2) glass/electrolyte solution; (3) solution/film (EA polymer); and (4) film/electrode. It was shown [55] that the influence of these interfaces on the obtained Raman results may be significantly reduced by adjusting the angle of laser beam incidence on the electrode, thus increasing the accuracy of the results interpretation. 

#### 2.2.1. Estimating Changes in Metallic Electrode Reflectance

The EA polymer film usually changes its absorption of the laser radiation during the electrodeposition or redox doping/dedoping. This results in the changing degree of doubling of the polymer film excitation by the sum of the incident and reflected laser beams, which distorts the results of quantitative analysis of the changes in the polymer chemical structure. Recently [56,57], we have proposed a method to estimate the change in the degree of excitation doubling by using the intensity of water OH-stretching vibration near 3440 cm^−1^ as a measure of the decrease of the reflection beam intensity. 

**Figure 6:**
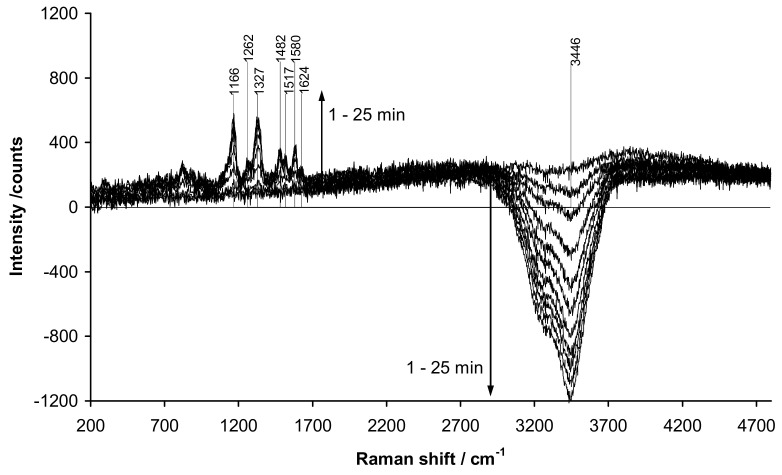
Evolution of Raman spectra (532 nm excitation) of Pt-electrode during galvanostatic electropolymerization of aniline in aqueous solutions of 0.1 M aniline in 0.5 M H_2_SO_4_. Reprinted from Journal of Electroanalytical Chemistry Volume 873, A.A. Nekrasov et al., “Raman spectroelectrochemical monitoring of conducting polymer electrosynthesis on reflective metallic electrode: Effects due to double excitation of the electrode/film/solution interfaces”, Pages No. 114415 (1-12), Copyright 2020, with permission from Elsevier [56].

For example, in the case of aniline electropolymerization [56] the initial spectrum of Pt electrode in solution, which is measured at conditions of double excitation and is stored as background, contains the maximum intensity of vibration of the band of water. As the electrosynthesis proceeded, alongside the growth of specific vibrations of PANI in the range of 1000–1700 cm^−1^ we observed gradual appearance of the negative Raman amplitude in the range near 3440 cm^−1^ (see Figure 6) due to reducing reflection of laser beam from the Pt-electrode. It is clear that the amplitudes of the specific vibrations of PANI in the Raman spectra sequentially registered in these conditions cannot be quantitatively compared. However, one may apply the following correction procedure assuming that the intensity of water vibration should be constant during the electrosynthesis of PANI and there is a linear dependence between the excitation energy and amplitudes of PANI vibrations in the Raman spectrum: (1) in each minute of the electropolymerization one can calculate the ratio between the amplitude of water vibration in the initial solution (subtracted as a background) and the negative amplitude registered in this minute obtaining a coefficient characterizing the degree of reduction of laser beam reflectance (reduction of the total excitation intensity); (2) one can multiply by this coefficient each point of the PANI spectrum in the area of 1000–1700 cm^−1^. 

A similar procedure may be applied to reduce the influence of the polymer film absorbance on the total excitation energy during in situ Raman spectroelectrochemical studies of redox transitions in EA polymers. In this case, the choice of a basis for comparison (water O-H vibration with maximum intensity) depends on the type of polymer (the character of evolution of its absorption spectrum with potential) and the wavelength of laser used for Raman scattering excitation. For example, using 532 nm excitation for PANI one should take maximum water vibration intensity from the spectrum of the reduced form. On the contrary, for PEDOT film in the same conditions one should take maximum water vibration intensity from the spectrum of the oxidized form, which absorbs 532 nm laser radiation to a lesser extent. 

There is one more problem arising during Raman spectroelectrochemical monitoring of electrosynthesis of EA composite polymers–strong fluorescence in the thin layer of the synthesis solution before the electrode. For example, it was shown [57] that electropolymerization solutions containing aniline and polystyrene sulfonic acid (PSSA) under 532 nm laser excitation give intensive signals in a wide range of Raman frequencies, which overload the spectrometer. One may overcome this fluorescence by simple reduction of the laser excitation intensity or the integration time of the spectrometer; this will lead to significant loss of the sensitivity, and, therefore, accuracy of the spectra registration. Also, one may avoid fluorescence using laser excitation of longer wavelength (785 or 1060 nm), but this low-energy excitation decreases the intensities of high-energy characteristic Raman bands of PANI, and some of them may be not seen at all. Fortunately, a simple method was found [57] to solve this problem: to increase the angle of laser beam incidence on the electrode (up to 20°, perpendicular direction taken as 0°). As a result, the local volume of the electropolymerization solution in which the incident and reflected laser beams overlap (causing doubling of the excitation intensity) significantly diminishes and the intensity of fluorescent signal decreases. At the same time, the intensities of characteristic Raman bands of PANI observed against the background of the fluorescent signal reduced to a significantly lower extent.

**Figure 7:**
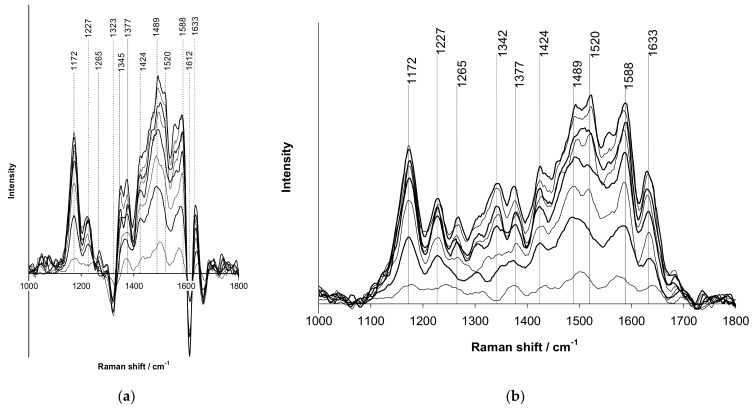
Evolution of the Raman spectra of PANI film during galvanostatic polymerization of aniline in the presence of t-PASA: (**a**) uncorrected, baseline subtracted; (**b**) corrected, baseline subtracted. Reprinted from Journal of Electroanalytical Chemistry Volume 873, A.A. Nekrasov et al., “Raman spectroelectrochemical monitoring of conducting polymer electrosynthesis on reflective metallic electrode: Effects due to double excitation of the electrode/film/solution interfaces”, Pages No. 114415(1-12), Copyright 2020, with permission from Elsevier [56].

Application of the above-described reflectance correction procedure is needed in the case when the electrolyte used for electrosynthesis or redox cycling of EA polymer has intense Raman bands in the range of characteristic frequencies of the polymer. This was demonstrated on the example of aniline electropolymerization in the presence of rigid-chain polyacid t-PASA. Here one should emphasize, that t-PASA solution, similarly to PSSA one, gives intensive fluorescent signal due to the presence of sulfonated phenyl rings. Figure 7a [56] shows the evolution of uncorrected (background subtracted) Raman spectra during electrodeposition of a film of PANI-t-PASA composite. 

As one can see from this figure, the uncorrected spectrum is significantly distorted by the presence of negative amplitudes near 1323 and 1612 cm^−1^. These frequencies correspond very well to the most intensive Raman bands registered in the aniline/t-PASA electropolymerization solution at the naked Pt-electrode and then stored as a background before starting the process. In the course of the electropolymerization due to decreasing reflectance of the Pt-electrode, the subtraction of stored t-PASA Raman bands causes the appearance of the negative amplitudes, similarly to the signal of water near 3440 cm^−1^ in Figure 6. The procedure to overcome this problem looks as follows: (1) background subtraction; (2) in each minute of the electropolymerization one calculates the ratio between the initial amplitude of water vibration near 3440 cm^−1^ and the negative amplitude of water registered in this minute to obtain a coefficient characterizing the degree of electrode reflectance change; (3) multiply by this coefficient each point of the initial spectrum of the species in solution in the characteristic area of 1000–1800 cm^−1^; (4) add the obtained value to each point of the characteristic Raman spectrum of the polymer film registered in this particular minute. As a result, one can obtain reasonable evolution of the Raman spectra of PANI film presented in Figure 7b.

#### 2.2.2. Confocal Raman Microscopy

Among the recent modifications of the Raman spectroscopy, one should obviously mention confocal Raman microscopy. However, we have found only two papers describing application of this method in spectroelectrochemical mode for PPy [58], poly(3-hexylthiphene) (P3HT) [59]. At the same time, this method becomes very useful if one needs to analyze the homogeneity of the distribution of the doping level or the components of polymer–polymer or polymer–inorganic blends within a compact layer. In this case the confocal Raman microscope can scan the surface of the layer and then special software builds a map of distribution of intensity of a characteristic vibration band along the surface. It was done to analyze the distribution of doping level of thiophene derivatives [40], of carbon nanotubes (CNT) in PEDOT/PSS [60] or fullerene/polymer phases in bulk-heterojunction layers for organic solar cells [61,62].

### 2.3. In Situ Infrared Spectroscopy

In situ infrared (IR) spectroscopy was among the first physico-chemical methods applied to study structural transformations during redox transitions in EA polymers. For example, by analyzing Fourier Transform Infrared (FTIR) reflection spectra of PANI film on Pt-electrode it was shown [63] that main the product of hydrolytic degradation of PANI at high anodic potentials in aqueous medium is para-quinone. Thus, the mechanism of this process was confirmed. At the same time, the application of IR-spectroelectrochemistry in the transmission or reflection mode is complicated by the presence of an IR-absorbing electrolyte layer on the path of the probe beam of spectrometer. Also, in the IR-transmission mode there is the problem of choosing a proper IR-transparent electrode, which is usually solved by the application of metal-grid electrodes or a thin film of boron-doped diamond deposited on silicon [64]. The most convenient mode used for IR-spectroelectrochemistry is attenuated total reflection (FTIR ATR), in which metal-grid, boron-doped diamond or semi-transparent Au [65] electrodes are applied onto the side of ZnSe ATR crystal contacting with electrolyte solution. 

### 2.4. In Situ Electron Spin Resonance Spectroscopy

Electron spin resonance (ESR) spectroscopy is a very useful technique for understanding intermediate stages of electropolymerization or the redox doping/dedoping mechanisms of EA polymers. Most of them include the stage of radical cation (paramagnetic species) formation followed by their recombination (paramagnetic species). The most representative example of this process was described in [48], where a sharp peak of spin concentrations was observed for PANI in the range of potentials of the first redox stage (leucoemeraldine ↔ emeraldine). This is followed by ESR-silent range (recombination) between the first and second CV peaks and sharp peak of spin concentrations, yet of lower intensity, in the range of potentials of the second redox stage (emeraldine ↔ pernigraniline). In situ ESR spectroelectrochemical studies are usually carried out using metal grid electrodes [36]. Particularly valuable information one can get by coupling ESR and UV–Vis spectroelectrochemical techniques [66].

## 3. Spectroelectrochemical Approach to Study Electroactive Composite Materials

### 3.1. Spectroelectrochemistry during Electrosynthesis of Electroactive Polymers

In situ UV–Vis–NIR spectroelectrochemistry has proven to be a very useful analytical tool to analyze the formation of electronic structure of EA polymers during electrosynthesis. The earliest works were devoted to investigation of initial stages of the polymerization reaction employing in situ spectroelectrochemical techniques. They were presented by E.M. Genies [67], Łapkowski [68] and the group of S.-M. Park [69] for PANI, the group of S.-M. Park [70] for PPy.

A novel method to characterize the structure of electrodeposited EA polymers has been proposed in [71] on the basis of the evolution of the UV–Vis spectrum of pyrrole solution in the course of its oxidative electrolysis. The necessity of a proper subtraction of the contribution due to absorption by various oxidation products generated inside the electrolyzed solution has been revealed.

In [72], UV–Vis–NIR spectra were monitored in the whole wavelength range during Ppy electrosynthesis in acetonitrile solution with Bu_4_NBF_4_ as an electrolyte, and it was denoted that relatively larger absorbance values, reached in the spectral range above 600 nm, proved that PPy film is formed in its oxidized state. 

**Figure 8:**
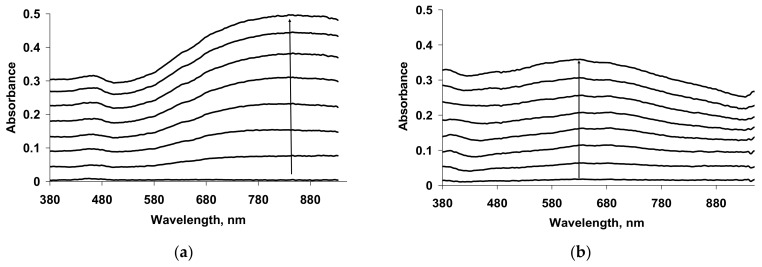
Evolution of UV–Vis–NIR absorption spectra of PPy films with time (increases in the direction of the arrows) in the course of electrosynthesis in GS-regime in the aqueous solutions of PSSA (**a**), i-PASA (**b**). Reprinted by permission from Springer Nature Customer Service Centre GmbH: Springer Nature, Journal of Solid State Electrochemistry, “Electrodeposition of thin films of polypyrrole-polyelectrolyte complexes and their ammonia-sensing properties”, O. L. Gribkova et al., Copyright 2020 [73].

Very similar spectral changes were observed in [73] for electropolymerization of pyrrole in aqueous solutions of H_2_SO_4_, Na_2_SO_4_ and flexible-chain sulfonated PE: PSSA and PAMPSA (Figure 8a). In the case of their sodium salts, as well as sodium salts of semi-rigid-chain poly-(4,4′-(2,2′-disulfoacid)-diphenylen-iso-phthalamide) (i-PASNa) and rigid-chain t-PASNa, a monotonic increase in the optical absorption was observed in the NIR region with a maximum near 730 nm. In the case of pyrrole polymerization in the presence of a semi-rigid polyacid i-PASA (Figure 8b), the absorption in the NIR region was much lower and the maximum (632 nm) was shifted to the shorter wavelength region. It was shown the acceleration of pyrrole electropolymerization in the presence of polyacids compared with the synthesis in aqueous solutions of H_2_SO_4_, Na_2_SO_4_ at the same concentrations of reagents [74].

**Figure 9:**
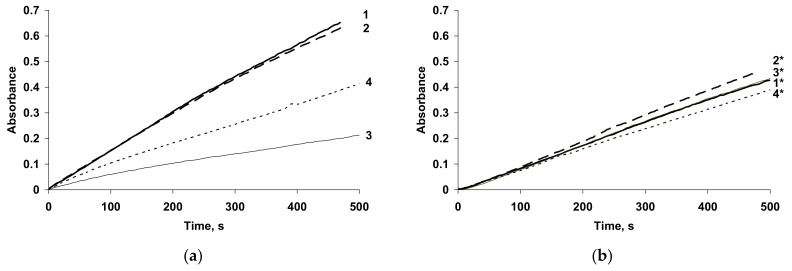
Time dependences of absorbance at 900 nm during GS synthesis of PPy in PSSA (1), PSSNa (1*), PAMPSA (2), PAMPSNa (2*), i-PASA (3), i-PASNa (3*), t-PASA (4) and t-PASNa (4*). Reprinted by permission from Springer Nature Customer Service Centre GmbH: Springer Nature, Journal of Solid State Electrochemistry, “Electrodeposition of thin films of polypyrrole-polyelectrolyte complexes and their ammonia-sensing properties”, O. L. Gribkova et al., Copyright 2020 [73].

The changes in absorption at 900 nm during galvanostatic (GS) electrosynthesis of PPy in all polyelectrolytes are presented in Figure 9. It is clearly seen that the absorbance transients are very similar for pyrrole electropolymerization in the salt forms of all PEs (Figure 9b) and t-PASA (Figure 9a, curve 4). The higher growth of absorption at 900 nm (Figure 9a, curves 1,2) is observed in the flexible-chain polyacids, indicating the formation of PPy films with higher electrical conductivity. The lowest absorption in NIR region was observed in the case of i-PASA and the growth of absorption slows down over time, indicating a decrease in the film electrical conductivity.

So, the investigation of PPy electrosynthesis by in situ UV–Vis–NIR spectroelectrochemistry allowed the elucidation of the influence of the PE backbone flexibility and its form (acid or salt) on the course of pyrrole electropolymerization. It was confirmed that PPy complexes with the acid forms of flexible-chain polyelectrolytes were formed in protonated, more oxidized and more conductive state compared to the PPy complexes with the salt forms of flexible-chain PEs. On the contrary, the character of PPy synthesis in the presence of the acid form of rigid-chain PE differed from common pyrrole polymerization at low pH in inorganic medium. The spectra of such complexes, especially the PPy complex with i-PASA, had lower absorption in the NIR spectral region, which was indicative of their lower electrical conductivity. The pyrrole polymerization in the salt forms of all PEs had similar character.

The potentiostatic electrosynthesis of PEDOT in aqueous media, without addition to the solution of any kind of surfactant, has been studied by in situ optical spectroelectrochemistry in combination with electrochemical quartz crystal microbalance (EQCM) [75]. These studies have given information about the oligomers’ generation and chain propagation, growing of the polymer deposit and concomitant p-doping, and overoxidation of the polymer film. In [76], the influence of electrosynthesis potential on electronic structure of PEDOT during EDOT electropolymerization in microemulsions containing polyoxyethylene-10-laurylether as a non-ionic micellar surfactant was investigated by in situ UV–Vis spectroscopy. It was shown that at high potentials overoxidation of the polymer takes place. Nasybulin et al. [77] showed by UV–Vis–NIR spectroscopic studies that the composition of codeposited poly(thieno[3,2-b]thiophene) and fullerene could be altered by changing potential range.

Tolstopyatova et al. [78] studied the influence of different supporting electrolytes on electrosynthesis of PEDOT films in acetonitrile. It was noted that the nature of the anion dopant affected insignificantly the rate of the film synthesis. In the case of PEDOT electrosynthesis in aqueous solutions of Pes, the significant influence was shown of the structure, the flexibility of the main PE chain and the form of PE (acid or salt) on the rate and the electronic structure of PEDOT films obtained [79,80,81].

**Figure 10:**
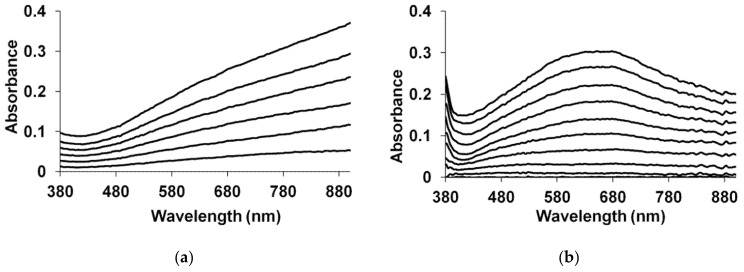
Growth of UV–Vis absorption of PEDOT films with time during GS synthesis in aqueous solutions of PAMPSA (**a**), t-PASA (**b**) [34].

The character of UV–Vis spectra evolution during electrosynthesis of PEDOT in the presence of salt forms of PEs irrespective of the structure, flexible-chain polyacids [80,81] and in inorganic electrolyte [75] was similar. For example, in the course of electrosynthesis in GS-regime in the aqueous solutions of flexible-chain PAMPSA (Figure 10a [34]) the intensive growth of absorption in the NIR-region testify that PEDOT is formed in bipolaronic state [82]. Significant differences in PEDOT spectra recorded during the syntheses were observed in case of using of more rigid-chain polyacids and a mixture of PAMPSA and t-PASA [34]. In this case the electropolymerization of EDOT was accompanied by the evolution of a broad maximum of absorption in the region of 600–700 nm (Figure 10b). The growth of absorption in this area is usually attributed to the polaronic form of PEDOT [82].

Thus, in case of EDOT electropolymerization in the presence of flexible-chain polyacids and salt forms of PEs and their mixture, the chemical structure of PEs molecules did not affect the course of PEDOT synthesis and electronic structure of the growing film. Oppositely, in the case of rigid-chain polyacids and mixture of rigid and flexible chain polyacid the formation of PEDOT films in polaron state was observed.

It should be noted that EDOT electropolymerization in 0.02 M H_2_SO_4_ was much slower, with a long induction period as distinct from the synthesis in polyacid-containing media. As a result, it takes ten times longer to achieve the same electrodeposition charge [79].

The course of aniline electropolymerization was studied by in situ spectroelectrochemistry also. Ibañez at al. [83] based on UV−Vis spectroelectrochemical monitoring during the elec-trodeposition of PANI and P3HT on each face of the FS-Single Wall CNT membrane concluded that Janus electrochemistry should be useful for the independent modifica-tion of the two faces of conductive membranes with different materials.

In [84], the electrochemical synthesis via cyclic voltammetry of PANI was studied by correlative electrochemistry and UV–Vis spectroscopy measurements in 1 M HCl. The authors showed that by combining the electrochemical processing and spectroscopic methods during the aniline polymerization, it is possible to correlate the PANIs’ oxidation and reduction processes (during CV cycling) with the corresponding PANIs’ electronic structures (correlation between the wavelengths and the energies of the electron transitions).

In [85], the in-situ UV–Vis monitoring during the electrosynthesis of poly(aniline-co-o-methoxyaniline) corroborated a strong formation of oligomers attributable to the methoxy group in the ortho position, which gave a much higher reactivity to the monomer. The reactivity damaged the electrical conductivity of the polymer, due to the occlusion of oligomers of N-phenyl-1,4-benzoquinonediimine.

**Figure 11:**
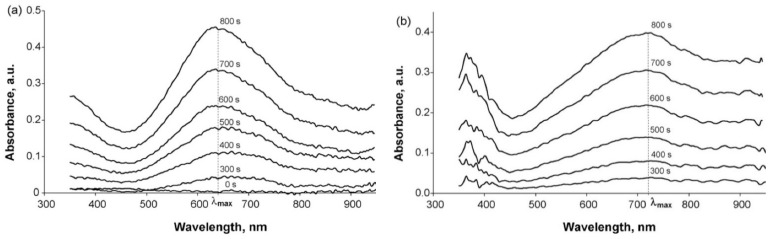
Typical evolution of absorption spectra of polyaniline film during the electrosynthesis at 0.75 V in the presence of: (**a**) flexible-chain polyacid (electrochemical bath: 0.0375 M aniline + 0.075 M PAMPSA; *λ*_max_ = 640 nm), (**b**) rigid-chain polyacid t-PASA (electrochemical bath: 0.0375 M aniline + 0.0375 M *t*-PASA; *λ*max = 720 nm). Reprinted from Electrochimica Acta Volume 53, A.A. Nekrasov et al., “Electrochemical synthesis of polyaniline in the presence of poly(amidosulfonic acid)s with different rigidity of polymer backbone and characterization of the films obtained”, Pages No. 3789–3797, Copyright 2008, with permission from Elsevier [86].

Typical evolution of electron absorption spectra of PANI films in the course of electrosynthesis in the presence of PAMPSA is presented in Figure 11a [86]. It is characteristic for aniline electropolymerization in aqueous solutions with inorganic acids [2,87]. The growth of absorption maximum at 640 nm is clearly seen. During aniline polymerization in the presence of more rigid-chain polyacids (Figure 11b) this maximum is shifted to longer wavelength and the absorption in NIR region is higher [86].

**Figure 12:**
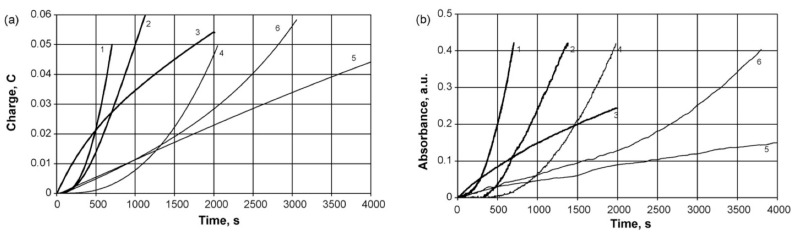
Time dependencies of the charge (**a**) and absorbance at λ_max_ (**b**) during the potentiostatic synthesis of polyaniline in aqueous solutions containing PAMPSA and HCl at 0.75 V vs. s.s.c.e. at different concentrations: 1: 0.0375 M aniline, 0.075 M PAMPSA; 2: 0.025 M aniline, 0.05 M PAMPSA; 3: 0.0125 M aniline, 0.025 M PAMPSA; 4: 0.0375 M aniline, 0.75 M HCl; 5: 0.0375 M aniline, 0.075 M HCl; 6: 0.0375 M aniline, 0.25 M HCl. Reprinted from Electrochimica Acta Volume 53, A.A. Nekrasov et al., “Electrochemical synthesis of polyaniline in the presence of poly(amidosulfonic acid)s with different rigidity of polymer backbone and characterization of the films obtained”, Pages No. 3789–3797, Copyright 2008, with permission from Elsevier [86].

The acceleration of PANI electrosynthesis in the presence of polyacids with different structure was clearly shown by using in situ UV–Vis monitoring during aniline electropolymerization at different concentrations of the synthesis medium [86]. The changes of electric charge (Figure 12a) [86] spent for the electrosynthesis and optical absorbance at the appropriate wavelength of maximum absorption (Figure 12b) [86] were compared. It should be emphasized that the shapes of the charge and absorbance dependences in the aqueous solutions PAMPSA and HCl were almost identical, which testifies to the fact that there is a good correlation between accumulation of the product absorbing at the chosen wavelength and charge spent for the electrosynthesis and the contribution of side reactions is insignificant. It should be noted that the polymerization rate in the presence of the polyacid (Figure 12a,b, curves 1) is an order of magnitude higher than that in the presence of an inorganic acid (Figure 12a,b, curves 4). In the presence of the polyacid the nucleation period is much shorter. This can be explained by preliminary association of aniline molecules along the sulfoacid groups, which favors the formation of PANI clusters near the electrode. Such clustering facilitates the nucleation of PANI at the electrode. The shapes of the curves for the polymeric and inorganic acids differ substantially, even if the volume concentrations of their protons are equal. For instance, autocatalytic polymerization (S-shape of the dependences) occurs in the presence of the polyacid (Figure 12a,b, curves 1), while with HCl the curve is far from being S-shaped (Figure 12a,b, curves 5). A tenfold increase in the concentration of the inorganic acid makes the curve S-shaped (curve 4). Also, a decrease in the concentration of the polymeric acid (Figure 12a,b, curves 3) reduces the induction period of nucleation and the polymerization is no longer autocatalytic.

In the presence of rigid-chain polyacid t-PASA the synthesis at all concentrations exhibits the autocatalytic character, while in the presence of semi-rigid-chain i-PASA, on the contrary, no induction period is observed and the growth is almost linear [86]. The explanations of this difference were based on the difference in rigidity of the polymer backbone of these two polyacids and irregular distribution of the sulfonic groups on the polymer chain. 

Raman spectroelectrochemical monitoring of PANI electrosynthesis comprises a complicated experimental task and there are few recent publications on this subject, for example [88,89]. However, by applying a correction procedure accounting for the change in reflectance of metallic electrode during the electrosynthesis (see Section 2.2.1), we have managed to prove that the formation of the radical cation form of PANI is the governing stage affecting the rate of aniline electropolymerization in the presence of sulfonic polyacids [56]. Later, the monitoring of PPy electrosynthesis in the same environment confirmed the adequateness of this approach [90].

PANI composites were electrosynthesized in the mixtures of flexible-chain PAMPSA and rigid-chain t-PASA [91]. On the base of spectroelectrochemical studies it was concluded that the rigid-chain polyacid had a predominant influence on the electronic structure of PANI composites with mixtures in the range of PAMPSA/t-PASA ratios from 6:1 to 1:6.

Thus, in situ spectroelectrochemical studies of electrosynthesis of EA polymers composites with PEs showed: (1) PEs act as molecular templates during electropolymerization accelerating the monomers polymerization and allowing to reduce concentrations of reagents compared to the synthesis in inorganic electrolytes; (2) the structure of PE influences the rate and character of the electrosynthesis, the electronic structure of the EA polymer films obtained. 

### 3.2. Spectroelectrochemical Studies of Redox Processes in the EA Polymer Films

#### 3.2.1. Basic Phenomena

The combination of different in situ methods provides important detailed information regarding some of the basic parameters characterizing the doping processes of EA polymers. For example, the simultaneous in situ ESR/UV–Vis spectroelectrochemical technique enables the monitoring of both ESR silent and paramagnetic species present in the investigated system during the redox cycling. The advantages of the in situ ESR/UV–Vis–NIR spectroelectrochemical measurements was presented in the study of PPy, polythiophene, oligothiophene and PANI [48,92,93,94]. 

Based on a combination of in situ spectroelectrochemical and in situ electrical conductivity measurements the ranges of potential, where conducting and isolating PEDOT phases exist, were determined [95]. It was implied that the electrical conductivity due to positive or negative polarons was of the same order of magnitude and that the higher maximum p-conductivity may be attributed to the generation of other charge carriers in the highly stable oxidized PEDOT films [96]. Simultaneously performed in situ conductance and spectroelectrochemical measurements proved [97] that fast redox switching can only be expected in cation exchanger type polymers, where the conductance develops promptly with the formation of mono-cationic/polaronic charge carriers, which are assumingly due to the presence of the charge compensating anions in favorable distribution. The redox transformation of PEDOT and poly(3-octylthiophene) had been studied and compared by combining simultaneous in situ UV–Vis–NIR spectroelectrochemical and ac impedance techniques [98]. 

Raman spectroelectrochemistry was first applied to study interpolymer complexes of PEDOT:PSS in [99,100]. Raman mapping has been used here to track “dynamic doping”, an important concept in organic electronics and in polythiophene-based solid-state electrochromic devices to understand and validate the mechanism of bias-induced redox-driven color switching [59]. 

Spectroelectrochemical mode of surface enhanced Raman scattering (SERS) was used for monitoring the redox states of PEDOT and poly(hydroxymethyl-EDOT) thin films in their potential application as oxidant detectors [101]. 

In situ FTIR ATR spectroelectrochemistry was used to study redox transformations in PEDOT thin films produced by layer-by-layer vapor phase polymerization at atmospheric pressure [102].

Bruchlos et al. [103] highlighted the important influence of film deposition conditions on the electrochemical and spectroelectrochemical behavior of P3HT. In [104] it was shown using Vis-NIR absorption, IR and Raman spectroscopy that the main carriers that were generated upon doping P3HT with FeCl_3_ solution were polarons. Upon doping with FeCl_3_ vapor, polarons also formed initially; at higher doping levels, bipolarons formed with the concomitant disappearance of the polarons. The Raman results indicated that the positive bipolarons were converted to polarons upon P3HT heating, indicating that the positive bipolarons formed a metastable state.

Nightingale et al. [105] developed in situ electrochemical resonant Raman spectroscopy. It was found that polaron formation in ordered P3HT polymer domains (crystalline phase) resulted in less pronounced changes in molecular conformation, indicating smaller lattice relaxation, compared to polarons generated in disordered polymer domains (amorphous phase) with large molecular conformational changes. The authors elucidated how blending the P3HT polymer with phenyl C-61 butyric acid methyl ester (PCBM) affected polaron formation in the polymer, by comparing well-mixed (as-cast) and phase-separated (thermally annealed) blends. The correlation between morphology of active layer and performance of polymer solar cells was investigated in [106] by spectroelectrochemical characterization of P3HT, PCBM cation and anion in varied active layers. Revealing the influence of polymer phase structures and the polymer-fullerene interface on the absorption spectra of the polarons was useful for understanding charge generation and transfer in polymer solar cell.

Zotti et al. [107] applied spectroelectrochemistry to polypyrrole and polyaniline for determining the polaron concentration and confirmed that polarons were stable intermediate species in these systems. The UV–Vis spectroelectrochemical properties of both free-standing films and PANI films deposited on ITO substrates were studied [108] at different electrolyte pHs, covering the range of 0.6 < pH < 3.0. It was shown that two species that could be associated to bipolarons and a polaron lattice are in chemical (not electrochemical) equilibrium with each other.

Spectroelectrochemical study was performed to represent the redox mechanism of PPy films [109]. Influence of the preparation conditions on the spectroelectrochemical properties of electrosynthesized PANI were discussed in [110]. Investigation of stability of reduced PPy by spectroelectrochemistry was presented in [111]. The degradation processes of the polymer film, as a function of the solution pH were studied by [54]. Solvent dependence of electrochromic behavior of PPy films was studied by spectroelectrochemical technique [112] and the effect of molecular oxygen on spectra changes was observed at very negative potentials.

Spectroelectrochemical properties of electrodeposited PPy, poly(N-methylpyrrole), polyindole and poly(pyrrole-indole) on gold and indium tin oxide (ITO) glass electrodes have been investigated and revealed their characteristic absorptions bands [113]. Based on optical spectroelectrochemical studies, ethylenedioxythiophene-based oligothiophene was characterized [114]. 

#### 3.2.2. Electrochromic Composite Materials Based on EA Polymer Films

In situ UV–Vis–NIR spectroelectrochemistry is a well-known characterization technique for electrochromic devises. It allows determining the main characteristics of electrochromic layers such as optical contrast, switching time and cycling stability. UV–Vis spectroelectrochemical technique was actively used to analyze the electrochromic properties of EA polymers in [115,116,117,118,119]. 

Two azo dyes, acid red 1 and acid red 18 [12] and three dye molecules of acid brilliant scarlet 3R, amido naphthol red G, indigo carmine, as well as sodium dodecyl sulfate as dopant agents [13] were used for electrochemical synthesis of PPy layers onto ITO electrodes. The investigation of spectroelectrochemical properties of the films showed that the simultaneous use of each dye molecule and the surfactant as dopant in PPy layers demonstrated proper electrochemical and optical stability and satisfactory electrochromic parameters. 

Films of PPy, PPy derivatised with methyl red (MR) and PPy doped with MR and the poly[3-(N-pyrrolyl)propyl-2-(4-dimethylaminophenylazo)benzoate] (PMRPy) were deposited onto ITO/glass [120]. The electrochromic properties of the PMRPy film, such as optical contrast, switching time and stability at the 100th cycle, were enhanced relative to the PPy/MR and PPy films. Tavoli et al. [14] presented the electrosynthesis and the spectroelectrochemical characterization of eriochrome cyanine doped PPy and showed that the electrochromic properties of dye-doped polymer were greatly enhanced relative to the PPy film. 

The interactions between polypyrrole and an embedded bromophenol blue (BPB) dye at different oxidation levels and pHs were investigated using a combination of cyclic voltammetry, in situ UV–vis and Raman microscopy techniques [58].

The electrochromic properties of Prussian blue–polypyrrole composite films in dependence on parameters of synthetic procedure was investigated by spectroelectrochemistry [121]. An indigo-carmine-doped PPy embedded with gold nanoparticles was synthesized by Loguercio et al. [122]. The studies of the effect of embedded gold nanoparticles on the electrochromic properties of the material showed the formation of a nanocomposite presenting enhanced electrochromic and optical properties, higher electroactivity and 10% lower band-gap energies, a two-fold increasing in the optical contrast and better optical stability.

The composite films consisting of PANI/sodium molybdate, and PANI/ferric nitrate were electrodeposited in oxalic acid aqueous solution and their electrochromic properties were compared [123]. UV–Vis spectrophotometric studies indicated the multiple color changes (yellow; green; and bluish green) in the doped films and that they had high absorption of UV radiation with respect to pure PANI films. The in situ UV–Vis spectra for PANI and polyaniline/titanium dioxide, polyaniline/zinc oxide were studied by Arjomandi et al. [124]. The results showed the intermediate spectroscopic properties between PANI and polymer nanocomposite films. 

Almtiri et al. [125] have prepared two PANI derivatives that contain a phenoxazine unit copolymerized with 2,5-dimethyl-p-phenylenediamine and p-phenylenediamine and determined their electrochromic properties. PANI nanofilaments were obtained by electropolymerization of aniline through the vertically aligned silica nanochannels **[126]**. The spectroelectrochemical data indicated more complete interconversion between the colored oxidized form and colorless reduced PANI for the arrays of nanofilaments in comparison to bulky films. In addition, the template-free nanowire arrays were characterized by faster electrochromic behavior than the PANI/silica hybrid. 

A rainbow multielectrochromic copolymer based on 2,5-di(thienyl)pyrrole derivative bearing a dansyl substituent and 3,4-ethylenedioxythiophene was obtained and its spectroelectrochemical properties were studied in [127]. Polythieno[3,2-b]thiophene with multicolor conversion via embedding EDOT segment was electrosynthesized [128]. Polymer possessed extended π-conjugation and narrowed band gap in molecular level. It achieved the mutual conversion between RGB primary colors (red–green–blue) under variable voltages. 

In [129] multilayer (PPy/PEDOT) electrochromic films were prepared. The results showed that different color options may be obtained by using layer-by-layer electrodeposition techniques. In [130] a multielectrochromic copolymer based on pyrrole and EDOT was successfully synthesized. P(Py-co-EDOT) film exhibited excellent multicolor electrochromism (amaranth, brick red, dark grey, and light blue). In [131] multilayered structures (PPy/PANI–PEDOT, PANI/PPy–PEDOT) were synthesized and their electrochromic properties were studied.

#### 3.2.3. Electroactive Polymer Composites with Inorganic Nanomaterials

The using of optical spectroelectrochemical studies for characterization of PEDOT composites with WO_3_ [21] indicated the redistribution of the fractions of different oxidized forms (polarons and bipolarons) in PEDOT/WO_3_ films. In [22] the spectroelectrochemical behavior of PEDOT/GO composites were studied in an ionic liquid (1-butyl-3-methylimidazolium tetrafluoroborate) as well as in acetonitrile and aqueous electrolytes. The composite film fabricated in water had its absorbance maximum at slightly higher wavelengths, and the color of the film was changed from well-known light blue one of PEDOT to grayish. The electrochromic characteristics of a number of composites of PEDOT with different carbon materials were compared [23]. The successful covalent attachment, via copper(I)-catalyzed azide-alkyne cycloaddition, of alkyne-functionalized nickel(II) and copper(II) macrocyclic complexes onto azide (N3)-functionalized PEDOT films was reported by Rodriguez-Jimenez et al. [132]. The spectroelectrochemical studies revealed that nickel-containing films demonstrated more pronounced bipolaron absorption then PEDOT films with Cu.

P3HT nanocomposites with TiO_2_ nanotubes or ZnO-coated TiO2 (ZnO/TiO_2_) nanotubes were fabricated and the influence of the inorganic nanostructures on the optical properties correlated with their photoactivity were studied by Cai et al. [133]. 

In situ Raman spectroelectrochemistry was used to study the mechanism of Zn-ion intercalation/deintercalation in a Zn−PPy secondary battery in aqueous and bio-ionic liquid electrolytes [134]. This method allowed the development of proof that Zn intercalation/deintercalation in aqueous solution occurs by a two-step mechanism, whereas a single-step mechanism of Zn storage is observed in bio-ionic liquid−water mixture electrolytes. The interaction of the electrodeposited PPy in the presence of ferromagnetic nanoparticle-incorporated triazine dendrimer with vitamin B12 were investigated by UV–Vis spectroelectrochemistry [135]. In situ electrical conductivity measurements and in situ UV–Vis spectroscopy of the polypyrrole-based gamma aluminum oxide and gamma iron (III) oxide (g-Fe_2_O_3_) nanocomposites revealed improvements in electrical conductivity by about 0.4 order of magnitude compared to PPy films and differences in spectroscopic behavior [20]. The PANI/sodium molybdate and PANI/ferric nitrate composite films [123] and PANI/titanium dioxide, PANI/zinc oxide films [124] were studied as electrochromic materials.

In situ Raman spectroelectrochemistry in a microdroplet setup was used to study the processes of preparation and redox cycling of two-dimensional graphene/polyaniline supercapacitors [136]. Selective formation of polyaniline on the graphene was confirmed. Also, the redox cycling was shown to result in the formation of benzoquinone−hydroquinone defects in polyaniline, which increased the specific capacity of the film during the initial 200 cycles, the capacity remaining stable during further 2400 cycles.

FTIR ATR spectroelectrochemistry was used to study the interactions in composite of graphene oxide and polyazulene prepared by electropolymerization in ionic liquid [137]. The doping-induced infrared active vibrations of the composite were found at higher wavenumbers indicating shorter conjugation of polyazulene in the composite.

#### 3.2.4. Electroactive Polymer Composites with Large Organic Anions

There are a number of works devoted to electrosynthesis of EA polymers in the presence of large anions. PPy films doped by polyanion Heparin (Hep) were electrochemically synthesized. The effect of different solvents (water, propylene carbonate, and acetonitrile) on the electrochromic features of electrodeposited polymers has been investigated by Alizadeh et al. [17]. PPy-Hep film exhibits high switching speed and the maximum transmittance contrast in water. In addition, presence of Hep causes drastic enhancement of electro-optical stability of PPy. In [138] Tiron-doped PPy film was electrochemically synthesized, and the effect of different deep eutectic solvents based on choline chloride ionic liquids on the spectroelectrochemistry and electrochromic features of electrodeposited polymer has been investigated and very significant solvent effect was found. 

In [15] PPy films were obtained in presence of a number of sulfonated dyes. The role of the sulfonated dyes on the spectroelectrochemical behavior of films was studied. It was shown that films modification with n-butylamine reduced polaron/bipolaron population and changed the electronic properties of the polymeric films. The spectroelectrochemical measurements showed that the potential-dependent spectra in PPy-Dye films were better than in PPy-ClO_4_ film after modification. Several dyes with different structures were used as dopant agents in electrochemical synthesis of PPy films for electrochromic application in [12,13,14,120,121,122]. The obtained composites enhanced the electrochromic properties of PPy films and increased the number of colors. 

Spectroelectrochemical results revealed differences both in the position of the spectral bands and their potential dependence for PPy and poly(pyrrole-2,6-dimethyl-β-cyclodextrin) films indicating interactions between polymer chains and β-cyclodextrin during electropolymerization process and possible decrease in chain length of the resulted polymer [16].

In the case of electrodeposited films of PPy composites with PE [139] the shape of spectra at fixed potentials was similar to the spectra of PPy films obtained in inorganic electrolytes [15,16,17,138] and the films exhibited similar spectroelectrochemical behavior. It should be noted that the spectroelectrochemical behavior of PPy films in the nonaqueous medium has the same tendency as in aqueous medium. Only a slight shift of characteristic maxima and isosbestic points to the short-wavelength region is associated with the change of solvent for spectroelectrochemical studies from water to organic medium [140]. 

**Figure 13:**
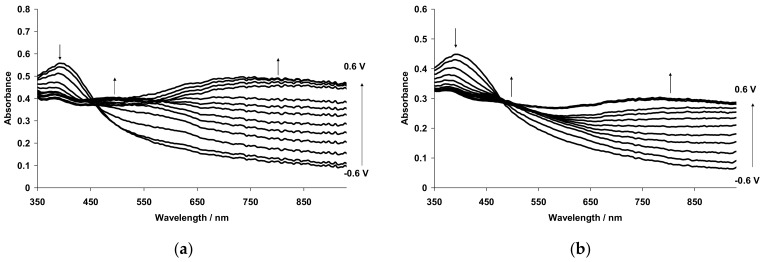
UV–Vis–NIR absorption spectra of PPy-i-PASA (**a**) and PPy-i-PASNa (**b**) films measured at different fixed potentials in an aqueous solution of 0.1 M NaClO_4_. Reprinted from Electrochimica Acta Volume 382, O.L. Gribkova et al., “Spectroelectrochemical investigation of electrodeposited polypyrrole complexes with sulfonated polyelectrolytes”, Pages No. 138307(1-14), Copyright 2021, with permission from Elsevier [139].

Figure 13 show the UV–Vis–NIR absorption spectra recorded at fixed potential for PPy-PE composites in an aqueous solution of 0.1 M NaClO_4_. PPy films have very similar optical UV–Vis–NIR-spectroelectrochemical properties [139]. At low potentials, the absorption band observed at about 400 nm is due to π-π * transition, which characterizes the reduced form of the PPy. With the increase of potential (oxidation), the intensity of this band decreases, while a weak absorption band is formed at about 500 nm, which is attributed to radical cations (polarons) and an increase of absorption in the NIR region of the spectrum (bipolarons) occurs.

The shift of all maxima and isosbestic points of PPy complexes with flexible-chain PEs in the salt form and Na_2_SO_4_ to the short-wavelength region compared to the complexes with acid forms of PEs and H_2_SO_4_ was explained by their lower protonation. 

At the same time, the spectroelectrochemical studies of PPy films in the UV–Vis–NIR regions in propylene carbonate containing 0.5 M NaClO_4_ ([139] Figure S8) showed that the dynamic range of changes in bipolaron absorption during spectroelectrochemical studies was higher in the case of PPy complexes with flexible-chain PEs. The number of cycles to achieve a 10% drop in the maximum dynamic range of absorption changes at 800 nm during cycling in the range of −0.3÷0.9 V was the highest for the complexes with the salt forms of rigid-chain PEs, followed by the salt forms of flexible-chain PEs ([139] Table 3). 

**Figure 14:**
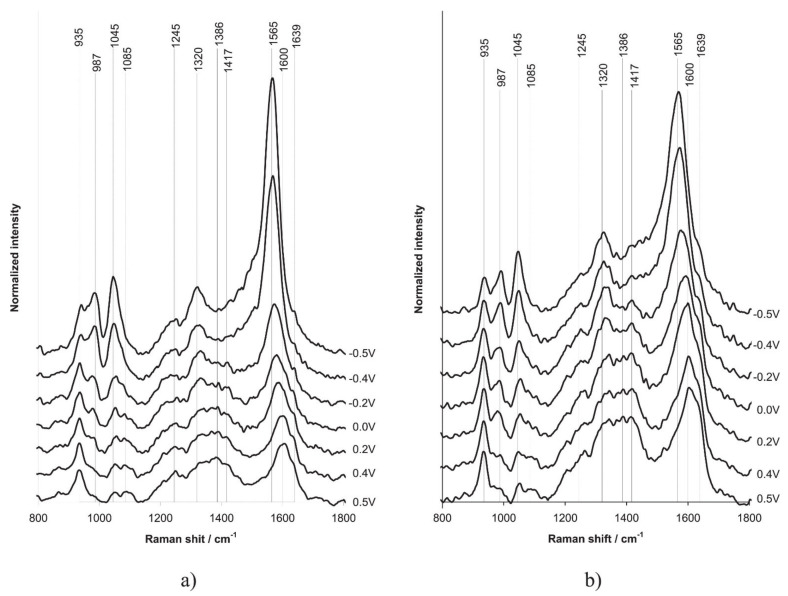
Normalized Raman spectra at 532 nm excitation for films of PPy complexes with PAMPSA (**a**), PAMPSNa (**b**), measured at fixed potentials in aqueous solution of 0.1 M NaClO_4_. Reprinted from Electrochimica Acta Volume 382, O.L. Gribkova et al., “Spectroelectrochemical investigation of electrodeposited polypyrrole complexes with sulfonated polyelectrolytes”, Pages No. 138307(1-14), Copyright 2021, with permission from Elsevier [139].

Using Raman spectroelectrochemistry it was shown (Figure 14), that introduction of different forms of polyelectrolytes into PPy film produces deep influence on their chemical structure. 

As one can see from Figure 14a, the vibration band near 1425 cm^−1^ characterizing the protonated form of PPy exists in the film of PPy-PAMPSA complex in all ranges of potential, as distinct from the PPy-PAMPSNa complex. This situation is also observed for the acidic forms of other polyelectrolytes.

There are a number of works describing electrosynthesis of PEDOT in the presence of large anions with unevenly distributed sulphonic groups. PEDOT/sulfated poly(β-hydroxyethers) composite films were prepared by electrochemical polymerization by Yamato et al. [141]. In situ absorption spectra of the composite films suggested that the composites with larger contents of PEDOT possessed shorter n-conjugation lengths. With increasing PEDOT content, the average distance between the neighboring sulfate groups on a polymer chain becomes shorter, thereby creating a more strained structure for both the PEDOT and the sulfated poly(β-hydroxyethers) chains in the composites. This gives rise to PEDOT polymers with shorter conjugation lengths. The shorter conjugation lengths of PEDOT electrodeposited in the presence of sulfonated polycatechol compared with PSSA was also stated in [18].

A number of works are devoted to spectroelectrochemical characterization of chemically prepared and then solution-cast PEDOT:PSS films [142,143]. In [142] CV with in situ conductance and in situ UV–Vis–NIR absorption spectroscopy measurements allowed for the following of the trends in conductance as a function of the charging level and to follow the evolution of the neutral, polaron (radical cation) and bipolaron (dication) species at the same time. The authors compared the solution-deposited PEDOT:PSS with ratios of 1:2.5 and 1:6. They have found that PEDOT:PSS(1:2.5) behave like classical conjugated polymers. In the case of PEDOT:PSS(1:6) an incomplete conversion of bipolaron and polaron states to neutral state was observed. It was explained by a high amount of PSS insulator that limits the inter-chain interaction between PEDOT moieties. It was shown that spectroelectrochemical properties of solution-deposited PEDOT:PSS(1:2.5) were the same as those of electrochemically deposited PEDOT film. In [144] PEDOT:PSS film was studied using steady-state and time-resolved spectroelectrochemistry. Careful deconvolution of the spectra using MCR analysis has allowed to separate the overlapping absorption signatures of the neutral, polaron and bipolaron states of PEDOT, so that their relative populations could be evaluated as a function of applied voltage and temperature. The authors concluded that redoping was faster than dedoping and both processes were rate limited.

**Figure 15:**
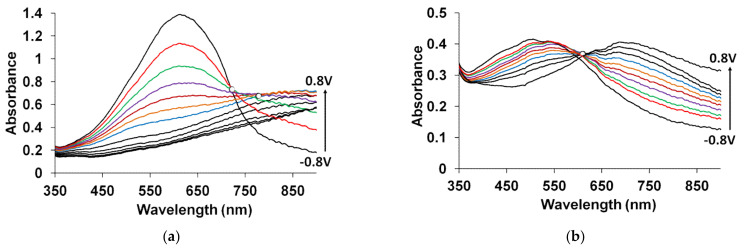
Optical absorption spectra of PEDOT films prepared in PAMPSA (**a**), t-PASA (**b**) measured at different potentials in 0.5 M aqueous solution of NaClO_4_. [34].

The spectroelectrochemical properties of PEDOT films obtained in such flexible-chain PEs as PAMPSA [79,80,145], PSSA(Na) [79,80,81,115,142,146] are very similar to those of PEDOT films prepared in aqueous or organic medium with small dopant ions [78,95]. Figure 15a shows the characteristic absorption spectra of PEDOT films at fixed potentials in the aqueous solution of 0.5 M NaClO4. A band near 600 nm at low potentials is due to π-π*-transitions in the reduced form of PEDOT. With an increase in the potential (oxidation), the intensity of this band decreases and, simultaneously, an absorption band at about 800–900 nm is formed (the polaronic form of the oxidized PEDOT). During this transition an isosbestic point appear near 710–720 nm. Further growth in the potential causes an increase in absorption in the NIR region of the spectrum (transition from polaronic to bipolaronic form) and the second isosbestic point can be observed. In reality the transitions from the neutral form to polaronic and then to bipolaronic one occurs in wider ranges of potential, but optical phenomena occurring during PEDOT doping/dedoping due to changes in the film thickness and its refractive index introduce some uncertainty in the isosbestic points’ positions (sometimes called “isosbestic range” [78]). At higher potentials the superposition of the 900 and 1550 nm bands makes it difficult to separate and to determine their relative contributions. In this context, Zozoulenko et al. [82] showed with the help of density functional theory calculations that electronic transitions due to polaron and bipolaron structures exist both at low and high oxidation levels, the polaron transition exhibiting a maximum at intermediate oxidation potentials. It should be noted that PEDOT films prepared in the presence of sodium salts of flexible-chain and rigid-chain PEs demonstrated very similar UV–Vis–NIR spectroelectrochemical behavior.

The character of changes in the electronic absorption spectra for PEDOT films prepared in rigid-chain polyacids (t-PASA and i-PASA) [79,80,81] and mixture PAMPSA/t-PASA [34] differed significantly (Figure 15b) from this one for traditional PEDOT films (Figure 15a). The maxima of the characteristic absorption bands of reduced (~500 nm) and polaron (~700 nm) fragments of PEDOT are shifted hypsochromically compared to those for PEDOT films obtained in the presence of PAMPSA and salt forms of all PEs and the mixtures. The observed shift to the short-wavelength region is due to a decrease in the length of the π-conjugation of the polymer chain and the formation of short PEDOT chains as it was observed in [18,141,142]. In addition, the retarded formation of a highly conductive bipolaronic form is observed, as can be seen from the low absorption in the NIR region. Such PEDOT films have only one “isosbestic range” shifted to the short-wave region (611 nm). Such differences were explained based on the main structural differences between the polyacids. The rigid-chain t-PASA and i-PASA with irregular distribution of sulfonic groups with short links to the main chain could not fully compensate double charges of bipolaron fragments on the rigid conjugated PEDOT due to steric hindrances. 

PEDOT films obtained in the mixtures of PAMPSA and t-PASA (even at twice higher content of the flexible-chain polyacid) demonstrated behavior similar to that of PEDOT-t-PASA film [34]. So, the rigid-chain polyacid t-PASA produced a dominating influence on the spectroelectrochemical properties of PEDOT-PAMPSA/t-PASA films. 

Due to the fact that the reduction of PEDOT composites with rigid-chain polyacids and mixtures of PAMPSA and t-PASA proceeded mainly via a single transition from the first oxidized state (polaronic) to the reduced state they demonstrated higher amplitudes of optical response to ammonia vapor than the composites with flexible-chain polyacids [10,34].

The spectroelectrochemical investigation of PANI composite with large immovable anions are discussed in literature also. Composites of PANI, poly(*o*-phenetidine) and poly(2-ethylaniline) with PSSA were prepared by electrochemical polymerization. UV–Vis spectra measurements showed the evidences for the dopped PANI systems to exhibit fast electrochemical response time, recorded at the temperature 298 K. The comparison of their electrochromic properties was presented in [147]. The potentiodynamic synthesis and subsequent characterization of PANI films grown in the presence of a water-soluble conducting polymer poly(2-methoxyaniline-5-sulfonic acid) (PMAS) was described in [148]. UV–Vis spectra of the PANI/PMAS film demonstrated that the composite had similar properties to polyaniline homopolymer. 

The UV–Vis–NIR spectra of PANI-PAMPSA composite film at fixed potentials are presented in Figure 1a. The spectra of PANI complexes with PAMPSA (or PSSA) and common PANI film have similar shapes, however, in the case of PANI-PAMPSA the maxima of characteristic spectra in the area 600–900 nm are shifted to the short-wavelength region by 20–30 nm compared to the corresponding spectra for PANI-HCl films [86]. This shift may be attributed to some specific degree of PANI protonation in the film doped with unmovable polymeric dopant. While increasing the potential of PANI-PAMPSA films up to 0.9 V we observe regular shift (similarly to ordinary PANI) of the absorption from the region near 800 nm to the area near 650 nm, indicating the formation of quinoid structures in PANI. 

In contrast to PANI composites with flexible-chain polyacids the results for PANI composites with rigid-chain polyacids are quite different (Figure 1b). The shift of absorption to the shorter wavelength area at high anodic potentials is very small in this case indicating that the formation of quinoid fragments is retarded. Also, the variation of absorbance with potential in the NIR region is far more intensive in this case as compared to PANI-PAMPSA films of similar thickness.

Such differences in the spectroelectrochemical properties of PANI composites (similarly to the composites of PPy and PEDOT) were connected to the specific features of macromolecules of polyacids: the chain flexibility and the distance between sulfonic groups. The presence of rigid-chain polymeric acids hindered the formation of bipolaronic structures in all EA polymers because the rigid polyacid molecules could not fully compensate all charged species in an EA polymer macromolecule.

PANI films prepared in the presence of PAMPSA/t-PASA mixtures (up to 6:1) had spectroelectrochemical behavior and high absorbance in the NIR region similar to the composites with rigid-chain t-PASA [91]. This fact demonstrates the dominating influence of the rigid-chain polyacid in the mixture

It is obvious that more intense modulation of the NIR-absorption is achieved by the film prepared in the presence of a rigid-chain polyacid. Moreover, ordinary PANI film exhibited a drop even in the NIR-absorbance at the potentials above 0.6 V. It is important that in the case of PANI-t-PASA film we observe synchronous change of the visible and NIR-absorption (as distinct from PEDOT, for which the increase of absorption in the NIR-range is accompanied by the decrease in the visible area). This fact is advantageous for using PANI-t-PASA composites in “smart” windows for light and heat fluxes control.

**Figure 16:**
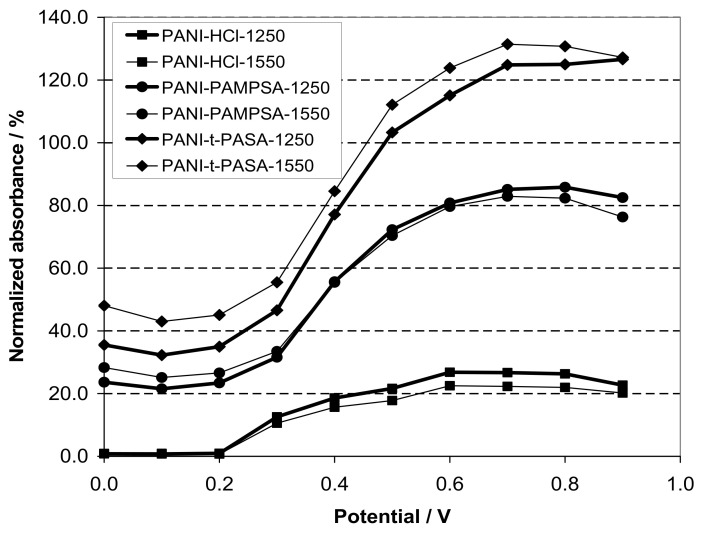
Changes of normalized (to the absorbance of localized polarons 750 nm) absorbance at 1250 and 1550 nm depending on the potential for the films of ordinary PANI and different composites. Reprinted by permission from Springer Nature Customer Service Centre GmbH: Springer Nature, Journal of Solid State Electrochemistry, “Electroactive films of interpolymer complexes of polyaniline with polyamidosulfonic acids: advantageous features in some possible applications”, A. A. Nekrasov et al., Copyright 2010 [149].

To estimate the efficiency of NIR-absorbance modulation for different films we have used the values normalized to the absorbance of localized polarons (750 nm) for each of them. Figure 16 presents the changes of normalized absorbance for standard PANI and the composites of PANI with flexible-chain and rigid-chain polyacids [149]. We can see the most intense changes occurring in the range of potentials 0.3–0.6 V. This is the evidence that the NIR-absorbing species probably have a radical-cation nature.

During investigations of the influence of PAMPSA molecular weight on the spectroelectrochemical properties of PANI-PAMPSA composite films, significant differences were detected [150]. As the molecular weight of PAMPSA was decreasing, the hypsochromic shift of PANI film absorption with increasing potential decreased and the appropriate absorption bands became less defined. Only slight differences between the shapes of spectra measured at 0.7 and 0.9 V were observed. The absorption maximum of the film at 0.5 V shifted to shorter wavelength region with the decreasing molecular weight of PAMPSA, indicating a reduction of the length of conjugation chain.

## 4. Conclusions

This review shows that in situ spectroelectrochemical techniques continue to play an important role in elucidating peculiarities of the mechanisms of synthesis and switching process in electroactive composite polymer materials under various types of chemical or electrochemical influence. The progress, in terms of the accuracy and reproducibility of the modern spectroscopic equipment, opens new prospects in application of these techniques. In parallel, new approaches to processing of the spectroelectrochemical data allows one to solve some problems with quantitative analysis of the obtained results.
